# Association Between Genetically Predicted Expression of TPMT and Azathioprine Adverse Events

**DOI:** 10.21203/rs.3.rs-2444787/v1

**Published:** 2023-01-13

**Authors:** Alyssa Davis, Alyson L. Dickson, Laura L. Daniel, Puran Nepal, Jacy Zanussi, Tyne W. Miller-Fleming, Peter S. Straub, Wei-Qi Wei, Ge Liu, Nancy J. Cox, Adriana M. Hung, QiPing Feng, C. Michael Stein, Cecilia P. Chung

**Affiliations:** Vanderbilt University Medical Center

**Keywords:** azathioprine, pharmacogenomics, thiopurine methyltransferase, rheumatology

## Abstract

Polymorphisms thiopurine-S-methyltransferase (*TPMT*) and nudix hydrolase 15 (*NUDT15*) can increase the risk of azathioprine myelotoxicity, but little is known about other genetic factors that increase risk for azathioprine-associated side effects. PrediXcan is a gene-based association method that estimates the expression of individuals’ genes and examines their correlation to specified phenotypes. As proof of concept for using PrediXcan as a tool to define the association between genetic factors and azathioprine side effects, we aimed to determine whether the genetically predicted expression of TPMT or NUDT15 was associated with leukopenia or other known side effects. In a retrospective cohort of 1364 new users of azathioprine with EHR-reported White race, we used PrediXcan to impute expression in liver tissue, tested its association with pre-specified phecodes representing known side effects (e.g., skin cancer), and completed chart review to confirm cases. Among confirmed cases, patients in the lowest tertile (i.e., lowest predicted) of TPMT expression had significantly higher odds of developing leukopenia (OR=3.30, 95%CI: 1.07-10.20, p=0.04) versus those in the highest tertile; no other side effects were significant. The results suggest that this methodology could be deployed on a larger scale to uncover associations between genetic factors and drug side effects for more personalized care.

## Introduction

Azathioprine is an immunosuppressive drug used to treat a variety of inflammatory conditions, including systemic lupus erythematosus (SLE), systemic vasculitis, and inflammatory bowel disease (IBD). The use of azathioprine is frequently constrained by side effects, for which a limited number of genetic predictors are known. One such example is dose-dependent myelotoxicity, a common and serious side effect of azathioprine use;^1,2^ variants in the gene encoding thiopurine-S-methyltransferase (TPMT), an enzyme in the metabolic pathway of azathioprine, can markedly increase the risk of myelosuppression. The TPMT findings, along with more recent findings regarding associations between variants in nudix hydrolase 15 (*NUDT15*) and azathioprine-induced myelotoxicity are now well established and have been incorporated into Clinical Pharmacogenetics Implementation Consortium (CPIC) guidelines for azathioprine dosing.^3^ However, many cases of myelotoxicity occur among patients with normal TPMT and NUDT15 metabolism;^4^ moreover, patients experience numerous additional side effects that can limit azathioprine use and burden patients, including rashes, malignancies, gastrointestinal intolerance, and infections.^5-7^ While genome-wide association studies (GWAS) have identified some of the genetic predictors of leukopenia, little is known about how emerging methods in pharmacogenetics can add to our knowledge of genetic factors that increase risk for azathioprine-associated side effects.

PrediXcan is a technique that estimates gene expression determined by an individual’s genetic profile through use of reference transcriptome data sets.^8^ Given that PrediXcan applies weights to estimate expression as a continuous variable and to account for the potential interaction between an individual’s larger genetic profile and a specific gene, this approach may potentially provide additional insights and increased power compared to assessments of SNPs as a categorical variable; as such, PrediXcan offers the possibility of identifying associations that have not been detectable previously using traditional GWAS or candidate SNP approaches. To assess the feasibility of using PrediXcan to identify associations between genetic markers and side effects of azathioprine, we performed a proof-of-concept study to determine whether the genetically predicted expression of TPMT or NUDT15 was associated with leukopenia or other known side effects of azathioprine use.

## Results

The cohort included 1364 new users of azathioprine. Patients were predominantly female (65.4%), their mean age was 44.7±17.6 years old, and they had a mean follow-up time of 3.1 ±3.8 years. Common indications for azathioprine included IBD (33.4%) and SLE (13.6%). There were no statistically significant differences in patient characteristics by tertile of TPMT expression (Table 1).

**Figure 1:**
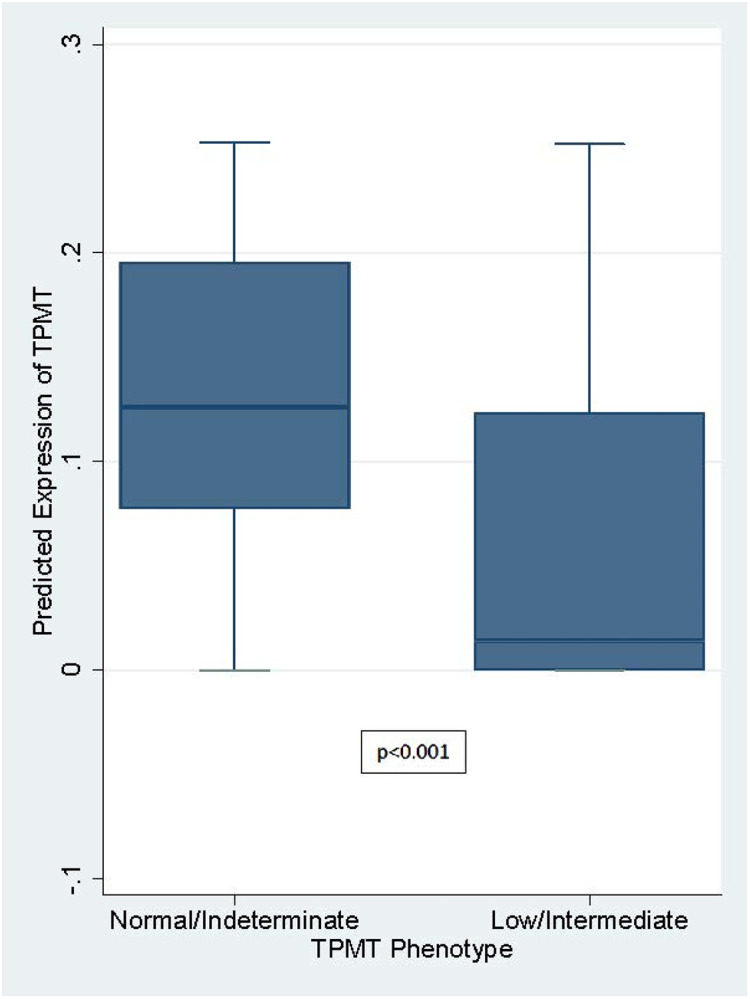
Predicted Expression of TPMT in Liver Tissue by TPMT Phenotype Group

There were too few individuals in this cohort with variance in *NUDT15*, either as a Clinical Pharmacogenetics Implementation Consortium (CPIC)-defined phenotype (n=9) or beyond a single node of predicted expression (**Figure S1**), for meaningful analysis. Indeed, we found no significant association between the CPIC-defined NUDT15 phenotype and the predicted expression of NUDT15 in liver tissue (p=0.40). In contrast, 105 individuals had variance in *TPMT*, as a CPIC-defined phenotype. The PrediXcan MASHR v8 model only contains one SNP in the TPMT liver tissue weight set (i.e., rs2842941); this SNP is not included in current CPIC guidelines for TPMT, nor is it in linkage disequilibrium with any SNP used in the CPIC guidelines on either a population basis or within the cohort (**Table S1**). Given the use of this single SNP, to validate the predicted TPMT expression in liver tissue, we first assessed the association between the predicted expression and predicted TPMT phenotypes, as determined by CPIC guidelines.^3^ Given the small numbers of poor (n=3) and indeterminate (n=4) metabolizers relative to intermediate (n=98) and normal (n=1259) metabolizers, we grouped the poor/intermediate phenotypes and normal/indeterminate phenotypes; the predicted expression of TPMT in liver tissue was significantly associated with these phenotype groups (p<0.001)(Figure 1). As a secondary step, we assessed the concordance between allele copies and predicted expression. While the three nodes of predicted expression were associated with the allele distribution (0, 1, or 2 copies of the rs2842941-T allele; p<0.001), there was overlap in predicted expression across the allele distribution, reflecting the impact of weights generated from individuals’ wider genotypes (**Table S2**).

Next, we tested the association of TPMT liver expression and the 51 phecodes representing known azathioprine side effects, using logistic regression (**Table S3**). Three phecodes had results with a pre-specified p<0.10: rash (n=40; p=0.01), non-basal cell skin cancer (n=13; p=0.02), and leukopenia (n=25; p=0.08). For each of the phecodes, we completed chart review of all possible cases to determine if the adverse event could be attributed to azathioprine. After clinical chart review, 25 (100% of possible cases confirmed) cases of leukopenia, 12 (92%) cases of skin cancer, and 4 (10%) cases of rash were found to be attributable (as described in Methods) to azathioprine use.

The predicted expression of TPMT had a trimodal distribution; therefore, when performing the analysis, we used tertiles of TPMT expression (**Figure S2**). When assessed by tertile of predicted TPMT expression, patients in the lowest tertile, representing those with the least TPMT expression, had higher odds of developing leukopenia (OR=3.30, 95%CI: 1.07-10.20, p=0.04) compared to patients in the highest tertile (Table 2). The results for skin cancer were not significant for the lowest tertile versus the highest tertile (OR=0.28, 95%CI: 0.06-1.36, p=0.12). The number of confirmed rash cases that were clinically attributable to azathioprine was too small for meaningful results. Results were consistent when adjusted by age, sex, or initial dose of azathioprine (Table 2).

We further interrogated the leukopenia findings to assess the utility of using this methodology for identifying potential genetic factors in azathioprine-related side effects. First, we assessed the leukopenia phecode for proficiency at identifying cases compared to laboratory results. We identified a total of 359 patients who had a white blood cell count < 4.0 K/μL during azathioprine use. All 25 patients with a phecode for leukopenia also had a white blood cell count measure < 4.0 K/μL, for a positive predictive value of 100% and a negative predictive value of 75%. We did not adjudicate the remaining 334 instances of lower white blood cell counts without a leukopenia phecode for non azathioprine-related explanations. Additionally, we examined whether the confirmed leukopenia cases occurred among normal metabolizers or individuals with compromised metabolism, per CPIC guidelines. Among the 25 phecode cases of leukopenia, 23 individuals were normal TPMT metabolizers and 2 were intermediate TPMT metabolizers. Finally, we assessed whether the use of the single SNP in TPMT used to derive the predicted expression was directly associated with the leukopenia phecode outcome. Relative to patients with the rs2842941-CC genotype, patients with the TC genotype had an OR=1.35 (95%CI: 0.43-4.32, p=0.60) and patients with the TT genotype had an OR=2.40 (95%CI: 0.74-7.72, p=0.14).

## Discussion

Our results show that genetically predicted expression of TPMT in liver tissue was associated with leukopenia among new users of azathioprine. Although we found no significant association between predicted TPMT expression and skin cancer or rash, this proof-of concept study suggests that PrediXcan may be useful for interrogating the association between gene expression and side effects of medications. In particular, PrediXcan may be useful for identifying other genes in the metabolic pathway of azathioprine. This finding may be especially helpful in identifying clinically significant genetic polymorphisms involved in drug metabolism.^9^ Identifying these genetic factors is important for the advancement of personalized medicine.

Known variants in genes encoding enzymes involved in the metabolism of thiopurines, such as TPMT and NUDT15, have strong effects and can help us predict whether a patient will have an adverse event.^3^ However, it is likely that many adverse events experienced by patients result from additional variants or the interaction of multiple variants.^4^ PrediXcan, particularly when paired with the use of phecodes, offers us a high throughput method to test the association between the cumulative effect of variants and multiple side effects. Indeed, given that 23 of the 25 cases of leukopenia detected by this approach had normal TPMT metabolizer status per current CPIC guidelines, this newly-identified predicted expression may provide clinical utility if added to those guidelines. Notably, the results were significant for the predicted expression only; while the results showed a similar pattern of increased risk when assessed by the SNP alone, these findings were not significant, suggesting that the predicted expression (i.e., weighted by an individual’s broader genotype) may be more informative for identifying certain genetic risk factors.

Numerous previous studies have demonstrated that individuals with the *TPMT* alleles that result in the inhibited metabolism of azathioprine have an increased risk of developing leukopenia while taking azathioprine, and this consensus is reflected in the CPIC guidelines for taking thiopurine drugs.^2,3,10^ We found that a reduction in predicted expression of TPMT was also associated with azathioprine-induced leukopenia. Given that the SNP used to predict TPMT expression in liver tissue was external to those used to develop CPIC guidelines, the findings suggest that the use of PrediXcan paired with phecodes may be a robust method for detecting previously unknown associations between predicted gene expression and adverse events, particularly if the impact of individual SNPs are not detectable by GWAS or are mediated by an individual’s larger genetic profile.

This study has limitations. First, GTEx models were validated in populations primarily composed of individuals with predominantly European ancestry.^11^ As such, we limited this analysis to White patients. We explored the possibility of expanding the cohort to include all races to test the feasibility of applying this methodology to patients with non-European ancestry, but the numbers were too small for any meaningful assessment; there were no additional cases of skin cancer, and only four potential cases of leukopenia. This also limited about ability to assess NUDT15 predicted expression. Second, and relatedly, the cohort size limited our power to detect significant differences in outcomes that were rare. Larger studies may provide further insight regarding the relationship between predicted expression of TPMT and skin cancer as well as allow the exploration of applying this methodology to patients with non-European ancestry. Third, our analysis was restricted to predicted expression in liver tissue, not other tissues. While this pre-specified choice seemed most appropriate given that metabolism of azathioprine is primarily hepatic, results may differ if performed using different tissues. Fourth, the phecodes may not be as sensitive for detecting side effects as directly gathering clinical data (e.g., laboratory results). Indeed, the results for rash suggest the potential difficulty of distinguishing between detecting azathioprine-associated side effects for diagnoses associated with azathioprine indications. Finally, the results did not reach significance after Bonferroni adjustment. Despite these limitations, this study provides proof-of concept that PrediXcan could help uncover novel associations between genes and adverse effects of medications that could impact clinical practice and outcomes.

## Methods

### Data Collection

This study was reviewed by the Vanderbilt University Medical Center’s (VUMC) Institutional Review Board and determined to be non-human subjects research (IRB # 180498). All methods were carried out in accordance with relevant guidelines and regulations. The cohort was derived from BioVU, a clinical practice-based biobank at Vanderbilt University Medical Center, a tertiary care center. BioVU uses de-identified electronic health records (EHRs)-including demographics, clinical notes, medical history, problem lists, medications, and diagnostic and procedure codes-which are linked with stored DNA samples.^12,13^ Informed consent to donate leftover blood samples from routine lab testing to BioVU for research was obtained from all subjects and/or their legal guardian(s) at time of lab collection.

For this retrospective cohort, we identified new users of azathioprine with at least one ICD9 or ICD10 code in their EHR during follow-up, as described below. We excluded patients whose genetic data did not pass quality control or whose primary indication for azathioprine was an organ transplant. Given that PrediXcan has been validated primarily in patients of European ancestry,^14^ we restricted the cohort to patients with EHR-reported White race; in previous work, we determined EHR-reported White race was highly concordant with European ancestry among new users of azathioprine in BioVU (i.e., >99% of patients identified as having White race had predominantly European ancestry).^15^

Chart review was completed to confirm azathioprine use and gather covariates. Patients entered the cohort on the first day of new azathioprine use, defined as no previous mention of azathioprine or mercaptopurine use in the EHR. Follow-up ended on the first of the following: 1) day of azathioprine discontinuation; 2) last confirmed azathioprine prescription or use as per the EHR plus 90 days; (3) lost to follow-up; (4) day of death; or (5) end of the study (December 31, 2018). We abstracted additional data from the EHR such as reported race, sex, age, initial daily dose of azathioprine, and indication.

As previously described,^4^ we genotyped patients using the Illumina Infinium Expanded Multi-Ethnic Genotyping Array (MEGA)^EX^ platform, imputed the results using Michigan imputation servers,^16^ and applied standard genetic quality controls. We then used PrediXcan to estimate the expression of TPMT and NUDT15 in liver tissue; we pre-specified liver tissue as the most appropriate target given that most of azathioprine metabolism is hepatic.^17^ We first assessed whether the cohort had sufficient variability in *TPMT* and *NUDT15* to deploy PrediXcan successfully and proceeded with the remaining steps accordingly. PrediXcan predicts gene expression in tissues by combining MASHR v8 GTEx weights with estimated dosages from the Michigan Imputation Server. We then confirmed that the MASHR v8 SNPs did not replicate SNPs used in CPIC guidelines nor were they in linkage disequilibrium. Given that the Michigan Imputation Server estimates dosage by summing the posterior probabilities of having the alternate allele in the haplotype, we anticipated that the predicted liver expression would have clusters of values.^8,18,19^

Using clinical pharmacology databases (e.g., Micromedex), the Food and Drug Administration package insert for azathioprine, and peer-reviewed publications, we compiled a non-exhaustive list of nine common types of adverse effects associated with azathioprine use: infections, gastrointestinal intolerance, hematologic toxicity, dermatologic symptoms, skin malignancies, non-skin malignancies, hepatotoxicity, constitutional symptoms, and pulmonary symptoms.^20-22^ We then identified 51 phecodes, a method for assigning phenotypes using ICD9 and ICD10 diagnosis codes in claims data, corresponding to these nine adverse event types (**Table S4**).^23^ We gathered ICD9 and ICD10 diagnosis codes for each patient during follow-up and designated patients with ≥2 non-consecutive instances of an adverse event type phecode as potential cases. After assessing whether any of these phecode groups were associated with TPMT or NUDT15 liver expression (described further below), we reviewed EHRs for adverse event types that had a pre-specified p<0.10, using clinical notes to confirm that the adverse effect could be reasonably attributed to azathioprine. We reviewed all potential cases for leukopenia, rash, and non-basal cell skin cancer. Leukopenia was defined as white blood cell count <4.0 K/μL, which was not be attributed to other causes. Cases of rash were limited to those with an ICD code for rash which was not be attributed to other causes (e.g., indication for azathioprine). Skin cancer cases were biopsy-proven or reported in dermatology documentation. Review was completed by a clinician using a standardized adjudication form (**Figure S3**).^24,25^ Ambiguous cases were subject to final adjudication by the senior author. All adjudications were completed blinded to genotype and predicted expression. We did not review charts for potential cases not identified by a phecode.

### Statistical Analysis

For demographic and clinical characteristics, we present categorical variables as numbers and percentages, and continuous variables as means and standardized differences. We used Chi-squared tests to compare categorical variables and Kruskal-Wallis tests for continuous variables.

We first assessed whether the predicted gene expression had sufficient variance for analysis (i.e., not clustered in one node) and the relationship between the SNP(s) used to generate the predicted expression and those included in definitions of CPIC metabolizer phenotypes; we used the Kruskal-Wallis test to assess the association between the predicted expression of TPMT in liver tissue and CPIC guideline-based TPMT metabolizer groups (i.e., normal/indeterminate and poor/intermediate) as described previously.^4^ We next analyzed the association between predicted expression of TPMT and the 51 phecodes corresponding to the adverse effects of azathioprine using logistic regression. Phecodes associated with adverse events with a p<0.10 were deemed “potential” associations and progressed to the next step of clinical review for case validation. Finally, we grouped the predicted expression of TPMT in liver tissue into tertiles and conducted logistic regression to assess the association between predicted expression and adverse events confirmed by review. We also completed logistic regressions adjusted by (1) age at baseline, (2) sex, and (3) initial dose of azathioprine for each of the phenotypes.

We gathered laboratory results during azathioprine use, including white blood cell counts. As such, to assess the completeness of the phecodes, we compared the incidence of leukopenia phecodes to instances of patients with a laboratory result of white blood cell count <4.0 K/μL, calculating positive and negative predictive values. Additionally, we assessed the TPMT metabolizer status of confirmed leukopenia cases, as defined by current CPIC guidelines, to ascertain whether any identified cases were among normal metabolizers (and could thus show additive clinical utility). Finally, we directly tested the underlying SNP(s) used to predict expression in liver tissue to determine any associations with significant outcomes in the primary analysis.

Analyses were conducted using STATA version 17.0 ^26^ and R version 4.1.0. ^27^

## Figures and Tables

**Table 1:** Baseline Characteristics of New Azathioprine Users by Tertile of Predicted TPMT Expression in Liver Tissue

	3 Tertiles of Estimated TPMT liver Expression			p- value
	Tertile 1 (N=455)	Tertile 2 (N=456)	Tertile 3 (N=453)	
**Female, n (%)**	290 (63.7)	299 (65.6)	303 (66.9)	0.61
**Prioritized Indication, n (%)**				0.14
**Systemic Lupus Erythematosus (SLE)**	38 (8.4)	43 (9.4)	42 (9.3)	
**Inflammatory Bowel Disease (IBD)**	145 (31.9)	148 (32.5)	162 (35.8)	
**Other Inflammatory Disease, not SLE or IBD**	242 (53.2)	243 (53.3)	238 (52.5)	
**Other**	30 (6.6)	21 (4.6)	11 (2.4)	
**Unknown**	0 (0.0)	1 (0.2)	0 (0.0)	
**Mean years of follow up, mean±SD**	3.1±3.8	3.2±3.8	3.1±3.7	0.99
**Mean age at initial dose, mean±SD**	44.4±17.6	44.8±17.6	44.9±17.5	0.91
**Initial Dose (in mg/day), mean±SD**	74.9±46.2	81.1±50.5	77.4±47.4	0.17
**Baseline WBC (in K/μL), mean±SD**	8.8±3.7	8.8±3.8	8.8±3.6	0.98

**Table 2:** Association Between Tertile of Predicted TPMT Expression in Liver Tissue and Confirmed Azathioprine-Associated Side Effects

	N Events	N At Risk	Unadjusted	Adjusted by Age	Adjusted by Sex	Adjusted by Initial Dose
***Leukopenia***						
Tertile 1	13	455	OR=3.30 (95%CI: 1.07-10.20), p=0.04	OR=3.32 (95%CI: 1.08-10.28), p=0.04	OR=3.32 (95%CI: 1.07-10.25), p=0.04	OR=3.29 (95%CI: 1.06-10.16), p=0.04
Tertile 2	8	456	OR=2.00 (95%CI: 0.60-6.70), p=0.26	OR=2.01 (95%CI: 0.60-6.71), p=0.26	OR=2.01 (95%CI: 0.60-6.72), p=0.26	OR=2.00 (95%CI: 0.60-6.70), p=0.26
Tertile 3	4	453	*	*	*	*
***Skin Cancer***						
Tertile 1	2	455	OR=0.28 (95%CI: 0.06-1.36), p=0.12	OR=0.29 (95%CI: 0.06-1.39), p=0.12	OR=0.27 (95%CI: 0.06-1.31), p=0.11	OR=0.28 (95%CI: 0.06-1.34), p=0.11
Tertile 2	3	456	OR=0.42 (95%CI: 0.11-1.64), p=0.21	OR=0.42 (95%CI: 0.11-1.63), p=0.21	OR=0.41 (95%CI: 0.11-1.62), p=0.21	OR=0.43 (95%CI: 0.11-1.66), p=0.22
Tertile 3	7	453	*	*	*	*
***Rash***						
Tertile 1	1	455	OR=1.00 (95%CI: 0.06-15.97), p=1.00	OR=0.99 (95%CI: 0.06-15.84), p=0.99	**	OR=0.98 (95%CI: 0.06-15.72), p=0.99
Tertile 2	2	456	OR=1.99 (95%CI: 0.18-22.04), p=0.57	OR=1.99 (95%CI: 0.18-22.01), p=0.58	**	OR=2.02 (95%CI: 0.18-22.35), p=0.57
Tertile 3	1	453	*	*	*	*

* Tertile 3 (highest predicted expression) is the reference.

** Sex is a perfect predictor of failure.

## Data Availability

Limited cohort data available by request to Dr. Chung at c.chung@vumc.org, pending BioVU approval and a data use agreement.
